# Assessment of satisfaction and Quality of Life using self - reported questionnaires after urethroplasty: a prospective analysis

**DOI:** 10.1590/S1677-5538.IBJU.2016.0207

**Published:** 2017

**Authors:** Eduardo Terra Lucas, Walter José Koff, Tiago Elias Rosito, Milton Berger, Tiago Bortolini, Brasil Silva

**Affiliations:** 1 Departamento de Urologia, Hospital das Clínicas de Porto Alegre, Universidade Federal do Rio Grande do Sul, RS, Brasil

**Keywords:** Urethral Stricture, Surveys and Questionnaires, Quality of Life, Cost-Benefit Analysis

## Abstract

**Objectives:**

To assess patient satisfaction and quality of life after urethroplasty using two different self-reported outcome measures and to compare it with objective clinical data.

**Materials and Methods:**

We prospectively collected data from 35 consecutive patients who underwent urethroplasty from January 2013 to September 2014. Patient demographics, International Prostate Symptom Score (IPSS), quality of life score, urethral stricture surgery patient-reported outcome measure (USS-PROM), maximum flow rate (Qmax) and post-void residual urine were collected before, two and eight months after surgery. Failure occurred when any postoperative instrumentation was performed. General estimation equation was used to compare the results and linear regression analysis to correlate both questionnaires with objective data.

**Results:**

Mean age was 61 years. Urethroplasties were equally divided between anastomotic and buccal mucosa grafts and 19 patients (59.3%) had a previous urethral procedure. Overall success rate was 87.5%. IPSS improved from a mean 19 at baseline to 5.32 at 8 months (p <0.001). The mean USS-PROM score also improved from 13.21 preoperatively to 3.36 after surgery (p <0.001) and 84.3% of patients were satisfied or very satisfied with surgical results. Mean Qmax increased from 4.64mL/s to 11mL/s (p <0.001). Strong negative correlation was found respectively between flow rate and USS-PROM (r=-0.531, p <0.001) and with IPSS (r=-0.512, p <0.001).

**Conclusions:**

Significant improvements in urinary symptoms and in quality of life are expected after urethroplasty and they are correlated with objective measures.

## INTRODUCTION

Urethral strictures are a high complexity disease that impacts on quality of life (QoL) with an increasing reported incidence in elderly population (1). The aim of any intervention is to restore patient’s normal pattern of voiding while maintaining a good QoL (2). Urethroplasty is considered the gold standard for the management of urethral stricture disease with excellent and durable successful reported rates (3, 4). It has already been shown that urethroplasty is the most cost-effectiveness strategy compared to minimally invasive procedures in the treatment of urethral stricture disease (5, 6).

The definition of what constitutes an urethroplasty success varies widely in the literature as well as the methods and frequency used to follow-up these patients. This lack of standardization makes comparisons between different studies difficult (7). Strategies of surveillance can range from subjective symptoms questionnaires to more invasive testing such cystoscopy and cystourethrography, that impact in the costs and the risks involved in this process. Despite reports that voiding symptoms have a good accuracy in predicting stricture recurrence, most outcome measures are clinician-driven indicators of technical success (8). Little has been published using patient-perceived symptoms and QoL outcomes after urethral reconstruction, although the recent development of a urethral stricture surgery patient - reported outcome measure (USS-PROM) is gaining considerable importance in the evaluation of patients’ perception of surgical success (9-11). The purpose of the present study was to prospectively analyze the pre- and post-operative patient-reported outcomes measures describing patient’s satisfaction and QoL after open urethral reconstruction and to compare these results with objective data.

## MATERIALS AND METHODS

We prospectively collected data from a cohort of consecutive patients older than 18 years old, Portuguese speakers who underwent urethroplasty from January 2013 to September 2014. The study was approved by the institutional review board of our hospital. Patients were excluded if they had not undergone formal reconstruction (i.e. perineal urethrostomy). Patients with previous open irreversible reconstruction, with evident cognitive impairment, or who refused to participate were also excluded. Patients lost to follow-up were excluded from analysis.

Preoperative evaluation included history taking for demographic characteristics. Stricture etiology, location and extension of the stenosis and previous treatments were collected. Retrograde and voiding cystourethrography were done preoperatively in all subjects to assess stricture length and site. If the patient had a suprapubic catheter for urinary diversion an anterograde cystourethrography was performed concomitantly to verify the urethral defect. The International Prostate Symptom Score (IPSS), QoL score of the IPSS, uroflowmetry, post-void residual urine (PVR) and the USS-PROM were collected preoperatively, 2 and 8 months after urethroplasty at the follow-up visits. The USS-PROM was developed in 2011 (11) as the first questionnaire specifically designed for patients with urethral stricture disease. This instrument is comprised of a LUTS domain and a health-related quality of life domain. The LUTS domain is composed by a six-item LUTS bother questions that generates a total score that varies from 0 (asymptomatic) to 24 (most symptomatic); by a separated LUTS-specific QoL question; and by the Peeling’s voiding picture, an illustration of a man voiding scored between 1 (best) and 4 (worst). The postoperative USS-PROM is supplemented with a treatment satisfaction question. The patients were invited to complete the LUTS domain and the overall satisfaction question.

All urethroplasties were performed by a single surgeon, and the surgical technique was at surgeon’s discretion. Urethral catheter was usually removed three weeks after surgery. Cystourethrography was done postoperatively only in patients who complained of urinary symptoms and was used to assess urethral patency and to direct further treatment if necessary. The surgery was considered a failure when any postoperative instrumentation or reoperation was performed.

The follow-up scores of IPSS, QoL, USS-PROM, Qmax and PVR were compared with preoperative scores and among them. The results of the IPSS and USS-PROM were also correlated with Qmax using linear regression analysis. To show an improvement of 10 points in the IPSS and 7mL/s in Qmax with a study power of 80% and p value <0.05, a sample size of 18 patients was estimated. General estimation equation was used to assess statistical significance between the baseline and postoperative time points. All statistical analysis was done using SPSS^®^ 18.0 with 2-sided significance considered at p <0.05.

## RESULTS

A total of 35 consecutive patients were included, of whom 3 were lost to follow-up and excluded from analysis. The mean age was 61 years (range 24-82). The average stricture length was 4.2cm (range 1-13) and strictures were located mainly in the bulbar urethra. The most commonly identifiable etiology was trauma in 11 patients (34.4%). In 16 patients (50%) a suprapubic urinary diversion was required due to complete urinary retention, thus obviating the preoperative assessment of subjective and objective data. A total of 18 (56.2%) substitution dorsal onlay repairs using buccal mucosa and 14 (43.7%) excision and primary anastomosis urethroplasties were done. Nineteen patients (59.3%) had undergone a previous urethral procedure. The overall success rate in the 8-month follow-up period was 87.5%, with all 4 urethroplasty failures presenting before the 2-month schedule visit with progressive worsening of voiding symptoms. Baseline data are summarized in Table-1.

**Table 1:** – Baseline characteristics.

Mean age, year (range)	61 (24 – 82)
Mean stricture length, cm (range)	4.2 (1 – 13)
	**n (%)**
**Stricture site**	
Penile	8 (25)
Bulbar	14 (43.7)
Bulbo-penile	4 (12.5)
Membranous	4 (12.5)
Panurethral	2 (6.2)
**Etiology**	
Trauma	11 (34.4)
Iatrogenic	7 (21.8)
Infectious	4 (12.5)
Idiopathic	10 (31.2)
**Suprapubic catheter**	**16 (50)**
**Procedure performed**	
Buccal mucosa graft	18 (56.6)
Anastomotic	14 (43.7)
**Previous Intervention**	**19 (59.3)**
DVIU	6 (18.7)
Dilatation	10 (31.2)
Urethroplasty	5 (15.6)

**DVIU =** Direct vision internal urethrotomy

Mean preoperative IPSS score was 19 and significantly decreased to 4.96 and 5.32 at the 2 and 8-months visit respectively (p <0.001). This small raise in IPSS in the follow-up period was not statistically significant. Patients also showed a significant improvement in QoL scores of the IPSS from 4.71 to 1.17 in the 2 and 8-month consultation (p <0.001). The mean improvements for IPSS and QoL score in individual patients at 8-months were respectively 13.64 (95% CI 18.14–9.13, p <0.001) and 3.43 (95% CI 4.20–2.65, p <0.001, Table-2).

**Table 2:** – Baseline to 8-month postoperative differences.

	Baseline Mean (95% CI)	8-mo Mean (95% CI)	P Value	Mean Difference (95% CI)	P Value
USS-PROM	13.21 (10.19 – 16.24)	3.36 (2.22 – 4.49)	<0.001	-9.21 (-12.70 to -5.71)	<0.001
IPSS	19 (14.45 – 23.55)	5.32 (3.87 – 6.78)	<0.001	-13.64 (-18.14 to -9.13)	<0.001
QoL	4.71 (4.05 – 5.37)	1.17 (0.71 – 1.85)	<0.001	-3.42 (-4.20 to -2.65)	<0.001
Qmax (mL/s)	4.64 (3.59 – 5.70)	11 (8.65 – 13.35)	<0.001	7.02 (3.22 to 10.77)	<0.001
PVR (mL)	41.04 (12.33 – 69.69)	5.07 (1.55 – 8.60)	0.017	-35.92 (-63.90 to -7.95)	<0.001

**CI =** Confidential Interval; **USS-PROM =** Urethral stricture surgery patient-reported outcome measure; **IPSS =** International Prostate Symptom Score; **QoL =** quality of life; **Qmax =** maximun flow rate; **PVR =** postvoid residual urine

The mean six-item LUTS score of the USS-PROM questionnaire was 13.21 at baseline, 2.46 two months after urethroplasty, and 3.36 eight months after urethroplasty (p <0.001). This worsening in LUTS score between the 2 and 8 month’s evaluation was not significant (p=0.193). The LUTS score showed a mean decrease of 9.21 points (95% CI 12.70–5.71, p <0.001, Table-2). The mean Peeling’s voiding picture score improved from 3.64 at baseline to 1.79 two months after urethroplasty (p <0.001). The 8-month score was 2.04 and was a trend towards worsening between the 2 and 8 month’s score, but without significance (p=0.064). The mean LUTS-specific QoL question was 3.21 at the preoperative evaluation and dropped to 1.36 at the 8-month follow-up (p <0.001). Overall 27 of 32 men (84.3%) of the patients were “satisfied” or “very satisfied” with the results of their urethroplasty at 8 months. From the 5 patients that were unsatisfied, 4 had recurrence of the stricture with worsening of their symptoms, requiring surgical reintervention.

**Figure 1:**
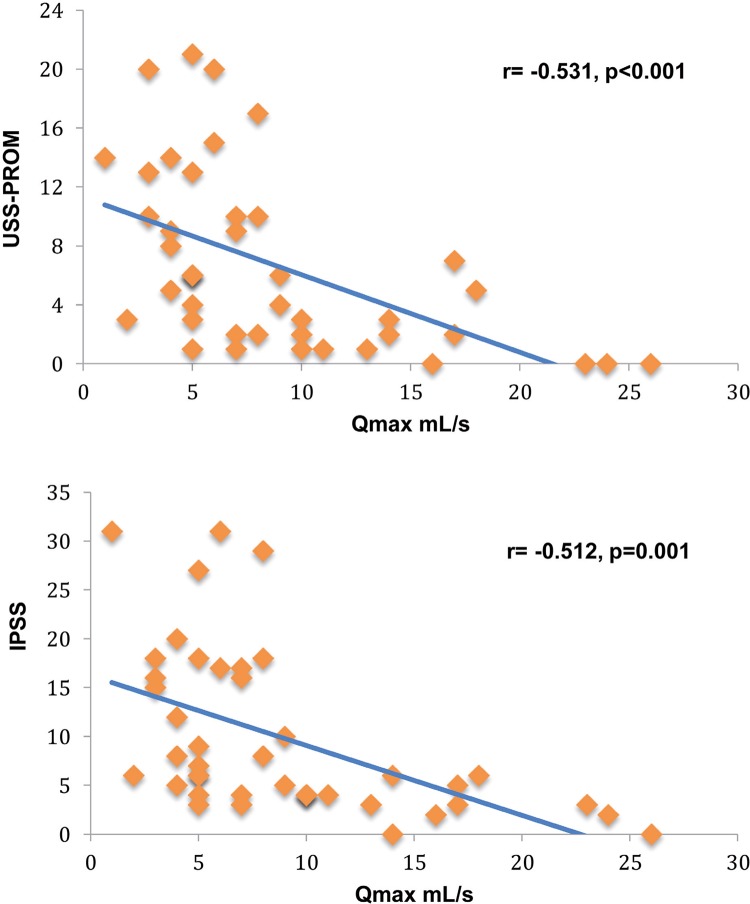
– Correlations between USS-PROM and IPSS scores with Qmax showing strong negative correlation.

The mean Qmax at baseline was 4.64mL/s and significantly increased to 11.11mL/s and 11mL/s, respectively at two and eight months (p <0.001). The mean improvement in Qmax from preoperative assessment to the 8-month evaluation was 7.02 (95% CI 3.22–10.77, p <0.001, Table-2). Mean PVR decreased from 41.04mL preoperatively to 5.07mL at the final visit of the study (p=0.017). The preoperative and postoperative USS-PROM and IPSS scores were compared with Qmax using linear regression analysis and a strong negative correlation was found for both (r=-0.531, p <0.001; r=-0.512, p=0.001, respectively; Figure-1). When patients were divided and analyzed by procedure type or by the presence of preoperative suprapubic urinary catheter, no significant differences were seen for IPSS, QoL, Qmax and USS-PROM in the post-urethroplasty assessment.

## DISCUSSION

This study presents patient-reported outcomes measures after urethroplasty using two different questionnaires for this purpose with each patient as his own control. We demonstrated continuous relief in IPSS, QoL and USS-PROM scores 8-months after urethroplasty with 84.3% of patients being satisfied or very satisfied with surgical results. For many surgeries the motivating factor is QoL, thus it is critical to know how satisfied patients are after undergoing urethroplasty both for counseling and following these patients up. Kessler et al. was among the first to highlight that subjective measures should be included in the assessment of urethroplasty outcomes (9). They noted that of 30 patients who were considered a failure by the surgeon’s perspective, 24 were subjectively satisfied or very satisfied with the surgical outcome, showing that patients may consider the outcomes differently than surgeons.

In addition to symptoms questionnaires we used uroflowmetry and PVR to evaluate surgical outcomes, and also demonstrated significant improvements after urethral reconstruction. Many investigators have used uroflowmetry to determine the success of urethroplasty, but usually do not correlate it with subjective findings (8, 12, 13). In our study, the mean Qmax improved from 4.64mL/s preoperatively to 11mL/s 8 months after surgery. These improvements in Qmax were more modest when compared with those reported in other series (12, 14). Maybe this occurred because our patients were older (mean age of 61 years), a group where a large number of men have a component of benign prostatic hyperplasia or have a long-standing urethral obstruction with detrusor dysfunction. Accordingly, DeLong et al. found a median improvement in Qmax of 12mL/s after surgery, but when splitting the cohort by age, patients with less than 45 years experienced an improvement of 16mL/s vs. 8mL/s achieved in those older than 45 years (15). Both studies highlight that the differences between the preoperative and postoperative data using the patient as his own control is, perhaps, more important than setting a Qmax cutoff at which all men should be evaluated for stricture recurrence. Differently, our self-reported outcomes represented by the USS-PROM, IPSS and QoL scores were not diminished by the lesser improvement in Qmax and the results were in accordance with the few studies that analyzed these questionnaires pre- and postoperatively (10, 14-16).

Monitoring patient symptoms should be a crucial part in any surveillance protocol for stricture recurrence. It is easy, readily available, cheap and with no adverse events reported. IPSS, although developed to assess treatments for benign prostatic hyperplasia, is the most frequently used questionnaire in the evaluation of urethroplasty outcomes (7). It was first used by Morey et al. who demonstrated significant improvements in IPSS after successful reconstruction and a strong negative relationship between the IPSS and Qmax (16), a finding also demonstrated in a subsequent study (13). Voelzke et al. in a systematic review of the literature examining the use of patient-reported outcome measures after anterior urethroplasties found only 4 articles that used a LUTS instrument (2). The development of a specific instrument to assess urethroplasty outcomes was necessary, a step made by Jackson et al., who in 2011 developed and validated a USS-PROM as an attempt to standardize patient-centered evaluations of interventions for urethral strictures (11). In 2013 the same group presented the first paper that prospectively evaluated the USS-PROM reporting continuous relief of patient’s symptoms and QoL in the 2-year follow-up period, setting a reference point which other groups can compare their performances with (10). The USS-PROM was also recently validated to Italian and German (17, 18).

Our cohort was only the second to report prospectively the results of the USS-PROM after urethroplasty and shows comparable outcomes. Overall 84% of our patients were satisfied or very satisfied with the results of urethroplasty at 8 months, compared to the 87% satisfaction rate reported by Jackson et al. (11). We found that the self reported six-item LUTS score significantly decrease postoperatively from 13.21 to 3.36 at 8 months, a better result than the 5.4 reported by the same group at a 2-year follow-up visit, but similar to the 3.4 score described by them at the 6-month survey. Moreover, we observed a strong negative correlation between the LUTS score and Qmax (r=-0.531, p <0.001), resembling the correlations described for Qmax and IPSS in other series (13, 16). The changes in Peeling’s voiding picture and in the LUTS-specific QoL question in our study also did not differ from that reported by Jackson.

To date the best strategy to evaluate stricture recurrence is not clear. Instead of this there are many different protocols varying from invasive testing such cystoscopy and voiding cystourethrography to non-invasive like questionnaire symptoms and uroflowmetry employed in surveillance regimens after urethroplasty. This is demonstrated by Meeks et al. who performed a meta-analysis and found an average of 3.15 different diagnostic tests for this purpose after surgery (19). This lack of standardization makes comparisons across different studies difficult as well as the ability to perform meta-analysis difficult, highlighting that follow-up protocols remains extremely variable.

Our study has some limitations, including that half of our population had a suprapubic tube, and this might have overestimated the results. We tried to minimize this effect using the General Estimation Equation in the statistical analysis. We also recognize that Qmax and especially PVR may not be enough to determine the anatomical success of the surgical repair. On the other hand, one of the objectives of our study was to correlate this non-invasive methods with subjective measures of success. For this purpose, further studies with more patients and longer follow-up are necessary. Additionally, the USS-PROM is not still formally validated to Portuguese.

In our practice, we use a multi-tier approach to screen patients after urethroplasty. We start with symptoms questionnaires, uroflowmetry and PVR comparing the results with the data collected preoperatively. If symptoms of voiding difficult or changes in uroflowmetry are present, we than proceed to more invasive evaluation. We found this strategy helpful in identifying recurrences while keeping patients comfort, favoring a less-expensive regimen. Furthermore, there is no evidence in the literature that early treatment of an asymptomatic recurrence is beneficial. Another issue of growing importance is the cost involved in the follow-up strategy in an era of increasing health care costs. Belsante et al. reported that a symptom-based, risk-stratified follow-up protocol would be far most cost-effective than close follow-up in all patients after urethroplasty, missing less than 1% of an asymptomatic recurrence (20). It has also been demonstrated that the first year charges of anterior urethroplasty surveillance can range from $205 to $1784 depending on the strategy adopted (21). It is our belief that urethral reconstruction is a quality of life surgery, and as long as the patient is satisfied with his symptoms, perform routinely invasive tests is, sometimes, overly aggressive, exposing patients to unnecessary risks and will usually not change management until patient feels symptomatic. The adoption of a disease-specific instrument like the USS-PROM questionnaire is of great value since it could help patients and physicians predict their outcomes and satisfaction.

## CONCLUSIONS

We have demonstrated that urethroplasty is a well-tolerated and worthwhile procedure by patient’s point of view. We have shown with both patient-reported outcomes and objective measures a significant improvement in symptoms, QoL scores and Qmax after urethral reconstruction using patients as their own control. Harmonization of surveillance protocols is clearly necessary as a method to more effectively compare the results between different reconstructive procedures and institutions. The use of a tool specifically designed to access urethral stricture disease is a great advance in this field and should be encouraged as an attempt to minimize costs and to incorporate the patients perspective in this process. Further studies are necessary to establish the optimal surveillance protocol for stricture recurrence and the place of PROMs in this setting.

## References

[1] Mundy AR, Andrich DE (2011). Urethral strictures. BJU Int.

[2] Voelzke BB (2013). Critical review of existing patient reported outcome measures after male anterior urethroplasty. J Urol.

[3] Barbagli G, Kulkarni SB, Fossati N, Larcher A, Sansalone S, Guazzoni G (2014). Long-term followup and deterioration rate of anterior substitution urethroplasty. J Urol.

[4] Andrich DE, Dunglison N, Greenwell TJ, Mundy AR (2003). The long-term results of urethroplasty. J Urol.

[5] Rourke KF, Jordan GH (2005). Primary urethral reconstruction: the cost minimized approach to the bulbous urethral stricture. J Urol.

[6] Greenwell TJ, Castle C, Andrich DE, MacDonald JT, Nicol DL, Mundy AR (2004). Repeat urethrotomy and dilation for the treatment of urethral stricture are neither clinically effective nor cost-effective. J Urol.

[7] Yeung LL, Brandes SB (2013). Urethroplasty practice and surveillance patterns: a survey of reconstructive urologists. Urology.

[8] Erickson BA, Breyer BN, McAninch JW (2010). The use of uroflowmetry to diagnose recurrent stricture after urethral reconstructive surgery. J Urol.

[9] Kessler TM, Fisch M, Heitz M, Olianas R, Schreiter F (2002). Patient satisfaction with the outcome of surgery for urethral stricture. J Urol.

[10] Jackson MJ, Chaudhury I, Mangera A, Brett A, Watkin N, Chapple CR (2013). A prospective patient-centred evaluation of urethroplasty for anterior urethral stricture using a validated patient-reported outcome measure. Eur Urol.

[11] Jackson MJ, Sciberras J, Mangera A, Brett A, Watkin N, N’dow JM (2011). Defining a patient-reported outcome measure for urethral stricture surgery. Eur Urol.

[12] Erickson BA, Breyer BN, McAninch JW (2011). Changes in uroflowmetry maximum flow rates after urethral reconstructive surgery as a means to predict for stricture recurrence. J Urol.

[13] Heyns CF, Marais DC (2002). Prospective evaluation of the American Urological Association symptom index and peak urinary flow rate for the followup of men with known urethral stricture disease. J Urol.

[14] Lumen N, Spiers S, Backer S, Pieters R, Oosterlinck W (2011). Assessment of the short-term functional outcome after urethroplasty: a prospective analysis. Int Braz J Urol.

[15] DeLong J, Buckley J (2013). Patient-reported outcomes combined with objective data to evaluate outcomes after urethral reconstruction. Urology.

[16] Morey AF, McAninch JW, Duckett CP, Rogers RS (1998). American Urological Association symptom index in the assessment of urethroplasty outcomes. J Urol.

[17] Barbagli G, Romano G, Sansalone S, Lazzeri M (2011). Italian validation of the English PROM-USS-Q questionnaire in patients undergoing anterior urethroplasty. Urologia.

[18] Kluth LA, Dahlem R, Becker A, Schmid M, Soave A, Rosenbaum C (2016). Psychometric validation of a German language version of a PROM for urethral stricture surgery and preliminary testing of supplementary ED and UI constructs. World J Urol.

[19] Meeks JJ, Erickson BA, Granieri MA, Gonzalez CM (2009). Stricture recurrence after urethroplasty: a systematic review. J Urol.

[20] Belsante MJ, Zhao LC, Hudak SJ, Lotan Y, Morey AF (2013). Cost-effectiveness of risk stratified followup after urethral reconstruction: a decision analysis. J Urol.

[21] Zaid UB, Hawkins M, Wilson L, Ting J, Harris C, Alwaal A (2015). The cost of surveillance after urethroplasty. Urology.

